# Transient Proteinuria Induced by High-Dose Rosuvastatin

**DOI:** 10.1016/j.aed.2025.05.003

**Published:** 2025-05-19

**Authors:** Khushi Goyal, Sandeep S. Soman, Arti Bhan

**Affiliations:** 1 Dayanand Medical College & Hospital, Ludhiana, Punjab, India; 2 Henry Ford Health System, Division of Nephrology, Detroit, Michigan; 3 Henry Ford Health System, Endocrinology Metabolism, Detroit, Michigan

**Keywords:** proteinuria, rosuvastatin, atorvastatin, statin-induced side effects, protein excretion

## Abstract

**Background/Objective:**

This case describes a 70-year-old woman who developed transient proteinuria after starting high-dose rosuvastatin following a non-ST elevation myocardial infarction. The objective of this report is to describe the development of proteinuria in a patient after high-dose rosuvastatin therapy and discuss the subsequent resolution with a medication switch.

**Case Report:**

A 70-year-old woman with a history of type 2 diabetes, primary hypertension, hypothyroidism, and hyperlipidemia, developed proteinuria after receiving high-dose rosuvastatin following an episode of non-ST elevation myocardial infarction. Prior to therapy, her low-density lipoprotein cholesterol was 123 mg/dL, coronary calcium score was 270, and urine albumin-to-creatinine ratios were 7.6 mg/g and 6.7 mg/g (normal albumin-to-creatinine ratio <30 mg/g). After 3 months of rosuvastatin therapy, proteinuria (albumin-to-creatinine ratio of 344.1) and muscle cramps developed, though her renal function remained stable (glomerular filtration rate >70 mL/min/1.73 m^2^). After discontinuing rosuvastatin and switching to atorvastatin (20 mg/d) and ezetimibe (10 mg/d), proteinuria resolved, and low-density lipoprotein cholesterol was maintained at 45 mg/dL.

**Discussion:**

Statin-induced proteinuria is a dose-dependent and typically reversible condition, more likely to occur with higher statin doses, such as rosuvastatin. Although proteinuria is generally transient, careful monitoring and dose adjustments are critical to optimizing statin therapy and patient adherence.

**Conclusion:**

This case highlights the importance of individualized statin therapy, emphasizing monitoring for dose-dependent side effects such as proteinuria.

**Highlights**

• High-dose rosuvastatin may cause transient reversible proteinuria without leading to permanent renal damage
• Clinicians should suspect statin-induced proteinuria in patients who are on potent statins, especially when they did not have proteinuria previously
• Statin therapy should be tailored to individual patients, considering individual risk factors and comorbidities
• Regular and close monitoring to see if proteinuria resolves or switching to another statin may be beneficial

**Clinical Relevance**

This case emphasizes the importance of individualizing statin therapy and monitoring for dose-dependent side effects like proteinuria, which can be managed with dose adjustments or alternative agents.

## Introduction

Statins are a cornerstone of cardiovascular risk reduction. Despite their proven efficacy, patients’ hesitancy due to concerns about side effects remains a challenge. Statin-induced proteinuria is a rare side effect of statin therapy, with studies suggesting a 1% to 2% incidence at typical doses.^1^^,^^2^ This case highlights a patient who developed transient proteinuria due to high-dose rosuvastatin, which resolved upon switching medications, underscoring the need for individualized treatment plans and close monitoring.

## Case Report

A 70-year-old woman with a history of type 2 diabetes mellitus (well-controlled on a glucagon-like-peptide-1 receptor agonist), primary hypertension (managed with an angiotensin-converting-enzyme inhibitor), hypothyroidism secondary to Hashimoto thyroiditis, and hyperlipidemia presented for cardiovascular risk assessment. She had an elevated low-density lipoprotein (LDL) cholesterol of 123 mg/dL, a coronary calcium score of 270 and urine albumin-to-creatinine ratios (ACRs) were 7.6 mg/g and 6.7 mg/g (normal ACR < 30 mg/g). Although, initially hesitant about starting statin therapy due to concerns about side effects, she agreed to begin low-dose rosuvastatin 5 mg/d after discussing risks and benefits. She tolerated this dose well for 2 months.

Following a non-ST elevation myocardial infarction, managed with early stenting and minimal myocardial damage, she received a single high-dose of rosuvastatin (40 mg) during hospitalization, which was then adjusted to 20 mg/d. Three months later, the patient developed proteinuria (ACR 344.1), confirmed on multiple tests. She also had muscle cramps that disrupted her sleep. Despite these symptoms, her renal function remained stable, with a glomerular filtration rate consistently above 70 mL/min/1.73 m^2^.

After a brief 2-day discontinuation of rosuvastatin, her muscle symptoms improved. She declined rechallenge with the medication and was transitioned to atorvastatin (20 mg/d) in combination with ezetimibe (10 mg/d). This regimen led to resolution of proteinuria (urine ACR reduced to 0.7 mg/g) within 1 month, while maintaining her LDL cholesterol at 45 mg/dL (Table).

**Table:** Laboratory Parameters Before and After High-Dose Rosuvastatin Therapy

Parameters	Pre-rosuvastatin therapy	Post-rosuvastatin therapy	After switching to atorvastatin
LDL cholesterol (mg/dl)	123	45	45
Albumin-to-creatinine ratio (mg/g)	7.6 and 6.7	344.1 (3 mo post-MI) 311 (4 mo post-MI)	0.70
GFR (ml/min/1.73 m^2^)	79	88	_
Serum creatinine (mg/dl)	0.81	0.73	0.73

Abbreviations: GFR = glomerular filtration rate; LDL = low-density lipoprotein; MI = myocardial infarction.

## Discussion

Statin-induced proteinuria is considered a dose-dependent and reversible condition, most notably associated with high-dose rosuvastatin.^3^ The underlying mechanism involves inhibition of receptor-mediated endocytosis, a likely mechanism of protein uptake in proximal tubular cells. The proteinuria associated with statin drugs is thought to be the result of interrupting the synthesis of cholesterol in proximal renal tubular cells, leading to inhibition of reabsorption of normally filtered proteins.^4^ This is not a nephrotoxic effect but rather seems to be a functional effect. It is rapidly reversible after statin discontinuation and is considered a class-effect, though most frequently observed with rosuvastatin in doses exceeding 20 mg/d.^5^

In this case, the temporal relationship between no proteinuria on low-dose statin; development of proteinuria on initiation of high-dose rosuvastatin and its resolution upon discontinuation of the medication is well evident. Differential diagnoses, such as diabetic nephropathy and hypertensive nephropathy, were unlikely given the patient's well-controlled diabetes, stable blood pressure, and absence of prior proteinuria. She did not have obesity, collagen vascular diseases, or the use of medications typically associated with proteinuria. Creatine phosphokinas was not checked in this patient, as her muscle symptoms were mild and did not warrant further investigation. Since the proteinuria resolved with the cessation of rosuvastatin, further extensive investigations were not deemed necessary. Patient declined rechallenge with the medication. To support the association between rosuvastatin and proteinuria in this case, the Naranjo Adverse Drug Reaction Probability Scale was applied. The patient scored a total of 7, indicating a “probable” adverse drug reaction.

A study by Shin et al (2022), also reported a 1.0% incidence of proteinuria in new users of rosuvastatin, with the risk increasing with higher doses (hazard ratio, (HR) 1.17, 95% CI 1.10-1.25). Notably, the study also highlighted a greater risk of proteinuria in patients with severely reduced kidney function (eGFR < 30 mL/min/1.73 m^2^), underlining the need for vigilant monitoring, particularly in high-risk populations such as those with chronic kidney disease or diabetes.^6^ Notably, although the incidence of nephrotoxicity is low, some case reports have raised concerns about direct renal tubular toxicity, particularly at higher doses.^7^ Despite these concerns, the overall risk of significant renal damage with statins, even at high doses, remains low, and long-term studies have demonstrated no permanent renal impairment. Statin therapy benefits in reducing cardiovascular risk far outweigh these transient adverse effects when the treatment is tailored appropriately.^8^ These findings align with our patient's case, where her renal function remained stable, as evidenced by a consistently normal glomerular filtration rate and serum creatinine. Moreover, our patient’s choice to switch to atorvastatin resulted in resolution of proteinuria and goal LDL reduction was maintained. Recommendations vary from monitoring patients closely to see if proteinuria resolves to switching to another statin. This approach ensures that any adverse effects are promptly identified and managed, without compromising the cardiovascular protective effects of statin therapy.

## Conclusion

This case illustrates the need for personalized statin therapy and vigilant monitoring for potential side effects such as proteinuria. Clinicians should suspect statin-induced proteinuria in patients who are on potent statins. When proteinuria occurs, adjusting the statin dose or switching to alternative agents can effectively resolve symptoms while achieving lipid-lowering targets.

## Statement of Patient Consent

Informed consent was obtained from the patient for publication.

## Disclosure

The authors have no conflicts of interest to disclose
